# Immune Escape by Non-coding RNAs of the Epstein Barr Virus

**DOI:** 10.3389/fmicb.2021.657387

**Published:** 2021-06-21

**Authors:** Christian Münz

**Affiliations:** Viral Immunobiology, Institute of Experimental Immunology, University of Zurich, Zurich, Switzerland

**Keywords:** T cells, NK cells, antigen processing, MHC presentation, humanized mice

## Abstract

Epstein Barr virus (EBV) is one of the most successful pathogens of humans, persistently colonizing more than 95% of the adult human population. At the same time EBV encodes oncogenes that can readily transform human B cells in culture and threaten healthy virus carriers with lymphomagenesis. Cytotoxic lymphocytes have been identified in experimental models and by primary immunodeficiencies as the main protective immune compartments controlling EBV. EBV has reached a stalemate with these cytotoxic T and innate lymphocytes to ensure persistence in most infected humans. Recent evidence suggests that the non-coding RNAs of the virus contribute to viral immune escape to prevent immune eradication. This knowledge might be used in the future to attenuate EBV for vaccine development against this human tumor virus that was discovered more than 55 years ago.

## Introduction on EBV and Its Oncogenesis

Epstein Barr virus (EBV) that is also called human herpesvirus 4 (HHV4) is a common human γ-herpesvirus establishing persistent infection in more than 95% of the human adult population (Münz, 2019). Characteristic for herpesviruses it exists in latent and lytic infection in its human host. Latent infection allows persistence, while lytic replication generates infectious viral particles for transmission. In the case of EBV, five latent infection programs, namely latency 0, I, IIa, IIb, and III, are thought to establish a latency reservoir in B cells for long-term persistence of this virus (Thorley-Lawson and Gross, 2004; Kempkes and Robertson, 2015; Table 1). For this purpose and after transmission by saliva exchange, EBV is thought to cross mucosal epithelia (Tugizov et al., 2013) for B cell infection in submucosal secondary lymphoid tissues like the tonsils (Farrell, 2019). In naïve B cells successively the latency IIb and III infection programs are established after EBV infection with the expression of all 6 nuclear antigens of EBV (EBNA1, 2, 3A, 3B, 3C, and LP), the small noncoding RNAs EBER1 and 2, 48 miRNAs in latency IIb and for latency III in addition the two latent membrane proteins (LMP1 and 2). The 48 EBV miRNAs originate from 25 miRNA precursors that are encoded in two clusters, namely the BHRF1 cluster (3 miRNA precursors) and the BART cluster (25 miRNA precursors) (Caetano et al., 2021; Pfeffer et al., 2004). The two latency programs IIb and III induce B cell transformation and latency III is also found in lymphoblastoid cell lines (LCLs) that can be generated by EBV infection of B cells *in vitro*. The associated B cell proliferation and rescue from apoptosis is thought to drive B cells into differentiation *in vivo* with successive down-regulation of viral transcripts (Babcock et al., 2000). In germinal center B cells only EBNA1, LMP1 and 2, EBERs and BART miRNAs are expressed (latency IIa). This latency can, however, be also reached without prior EBNA2 dependent latency IIb and III *in vivo* (Li et al., 2020). Latency IIa rescues infected cells from the germinal center reaction for persistence in memory B cells with only EBER and BART miRNA expression (latency 0) or additional EBNA1 expression during homeostatic proliferation (latency I) (Babcock et al., 1998; Hochberg et al., 2004). However, latency 0 persistence can be also achieved from latency IIb directly in the absence of EBNA3C (Murer et al., 2018). From latency 0 and I lytic reactivation of EBV can be induced by B cell receptor cross-linking, suggesting that cognate antigen exposure induces the immediate early transactivation factors BZLF1 and BRLF1 for lytic EBV infection (Kenney and Mertz, 2014). The resulting plasma cell differentiation is also associated with lytic EBV replication *in vivo* (Laichalk and Thorley-Lawson, 2005; McHugh et al., 2017). More than 80 early and late lytic gene products are then expressed together with EBERs and BHRF1 as well as BART miRNAs to produce infectious EBV particles (McKenzie and El-Guindy, 2015). Such lytic replication resulting in infectious particle production in submucosal secondary lymphoid tissues allows then for mucosal epithelia infection from the basolateral side (Tugizov et al., 2003; Chen et al., 2018; Zhang et al., 2018). Additional lytic replication in epithelia is thought to amplify shedding of EBV that is ideally suited for B cell infection and transmission via saliva exchange (Borza and Hutt-Fletcher, 2002; Hadinoto et al., 2009; Hutt-Fletcher, 2017). In addition to the EBER, BHRF1 and BART small ncRNAs, miRNA independent functions of the BART long ncRNA and EBV derived circular RNAs have been described (Bullard et al., 2018; Ungerleider et al., 2019). Among these the BART long ncRNA contains also a small nucleolar RNA that could be involved in RNA modifications (Hutzinger et al., 2009). However, their functions in EBV infected B cells and the regulation of latent and lytic infection remain largely unclear. Thus, EBV is ideally adapted to the human B cell physiology to achieve both persistence in long-lived memory B cells and lytic replication in plasma cells.

**TABLE 1:** EBV latency patterns.

Latency	0	I	IIa	IIb	III
Viral proteins	–	EBNA1	EBNA1, LMP1, LMP2	EBNA1, EBNA2, EBNA3A, EBNA3B, EBNA3C, EBNA-LP	EBNA1, EBNA2, EBNA3A, EBNA3B, EBNA3C, EBNA-LP, LMP1, LMP2
Viral small noncoding RNAs	EBER, BART miRNA	EBER, BART miRNA	EBER, BART miRNA	EBER, BART miRNA, BHRF1 miRNA	EBER, BART miRNA, BHRF1 miRNA
Associated tumors	–	Burkitt’s lymphoma, gastric carcinoma	Hodgkin’s lymphoma, nasopharyngeal carcinoma	–	Diffuse large B cell lymphoma, post-transplant lymphoprolifera-tive disease

**EBNA, EBV nuclear antigen; LMP, latent membrane protein; EBER, EBV encoded small RNAs, BART, BamH1 fragment A rightward transcript; BHRF1, BamH1 fragment H rightward transcript 1**

Despite asymptomatic EBV co-existence with its human host in most virus carriers, the above described EBV latency programs are, however, also associated with malignancies that occur more frequently during immune suppression after transplantation or human immunodeficiency virus (HIV) co-infection (Cesarman, 2014; Shannon-Lowe and Rickinson, 2019). In fact EBV causes around 200,000 tumors every year in the human population (Cohen et al., 2011). Latency I is found in Burkitt’s lymphoma and gastric carcinoma, latency IIa in Hodgkin’s lymphoma and nasopharyngeal carcinoma, and latency III in post-transplant lymphoproliferative disease (PTLD) and some diffuse large B cell lymphomas (DLBCL) (Shannon-Lowe and Rickinson, 2019). In this review I will discuss the role of EBV’s non-coding RNAs (ncRNAs), focusing on EBERs and miRNAs, in EBV driven tumorigenesis, transition of latent to lytic replication, and immune control thereof.

## Regulation of Viral Transcripts by EBV ncRNAs

EBER1 and 2 are non-polyadenylated RNA polymerase III transcripts of 162 and 172 nucleotide length, respectively (Farrell, 2019). Despite them being with several 1,000 copies per EBV infected cell (Lerner et al., 1981) among the most abundant viral transcripts and due to their expression in all latent and lytic infection program valuable for diagnostics by *in situ* hybridization (Weiss and Chen, 2013) EBER function still remains enigmatic. Both EBERs are not required for *in vitro* immortalization of human B cells and do not affect EBV infection of mice with reconstituted human immune system compartments (Gregorovic et al., 2011, 2015). Although deletion of EBER1 or 2 in recombinant EBVs leads to increased LMP2 mRNA in the respective LCLs immortalized with these viruses, LMP2 protein levels are not affected and no changes in the transformation frequency of primary human B cells or the growth of established EBER deficient LCLs were observed. Thus, EBERs seem to have little influence on viral gene expression.

**FIGURE 1:**
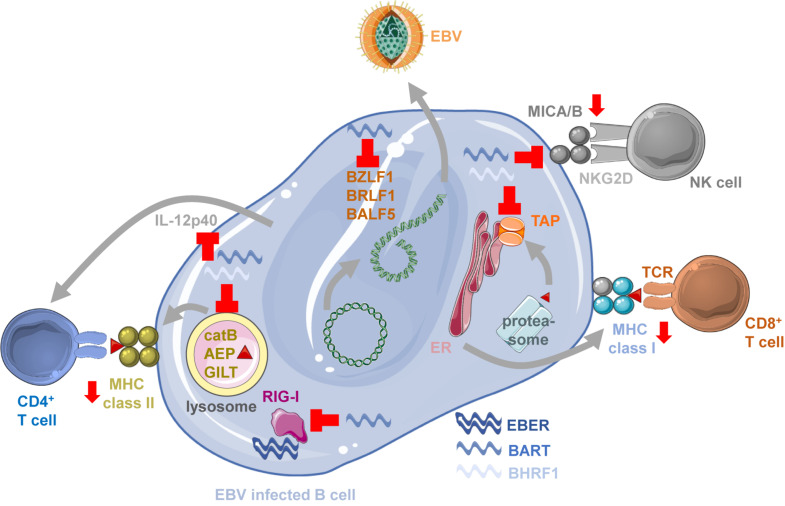
Immune modulation by EBV ncRNAs. BART miRNAs block lytic EBV reactivation by down-regulating BZLF1, BRLF1 and BALF5, limiting viral antigen production for immune responses. BART miRNAs block also MICA/B expression to inhibit NKG2D mediated NK cell recognition of EBV infected B cells. BHRF1 and BART miRNAs compromise antigen presentation by MHC class I molecules by down-regulating the transporter associated with antigen processing (TAP) to load MHC class I complexes with proteasome products in the endoplasmic reticulum (ER) for stimulation of CD8^+^ T cells. EBERs can be recognized by innate immune sensors of RNA like RIG-I, but the RIG-I pathway is also down-regulated by BART miRNAs. MHC class II ligand generation by lysosomal proteolysis is inhibited by BHRF1 and BART miRNAs down-regulating cathepsin B (catB), asparaginyl endopeptidase (AEP) and IFN-γ–inducible lysosomal thiol reductase (GILT). T cell priming and NK cell activation is also compromised by BHRF1 and BART miRNAs blocking IL-12p40 production. This figure was created in part with modified Servier Medical Art templates, which are licensed under a Creative Commons Attribution 3.0 unported license: https://smart.servier.com.

In contrast the RNA polymerase II miRNA precursors that are then processed by Drosha and Dicer, regulate both latent and lytic EBV gene transcripts (Skalsky and Cullen, 2015; Poling et al., 2017). Regarding latent EBV infection regulation it was originally noted that in the absence of BHRF1 miRNAs B cell transformation *in vitro* and EBV infection in mice with reconstituted immune system components is compromised (Feederle et al., 2011a,b; Wahl et al., 2013). Furthermore, LMP2 is down-regulated by BART miRNA 22 in nasopharyngeal carcinoma cells (Lung et al., 2009). In contrast to this regulation of latency establishment by the EBV miRNAs, BART miRNAs of EBV might be predominantly involved in blocking lytic replication (Figure 1; Cai et al., 2006; Chen et al., 2019; Serrano-Solis et al., 2019). Along these lines it was found that the BART miRNA 2 down-regulates the viral DNA polymerase BALF5 (Barth et al., 2008). This reduced BALF5 protein levels by 50% and infectious virus production by 20%. In addition, BART miRNA 20-5p down-modulates BZLF1 and BRLF1 transcripts (Jung et al., 2014; Lin et al., 2015). This diminishes lytic replication upon BART miRNA 20-5p expression. BART miRNAs are affected by the deletion that is present in the B95-8 laboratory strain of EBV (Yang et al., 2013) and EBV isolates with similar deletions are enriched in DLBCLs (Okuno et al., 2019). Therefore, it was suggested that enhanced early lytic EBV gene expression supports lymphomagenesis, probably via conditioning of the tumor microenvironment (Ma et al., 2011; Münz, 2019). BART miRNAs are expressed during all EBV infection programs, thereby presumably blocking this pro-tumorigenic effect of early lytic EBV gene expression. Furthermore, BART miRNAs are expressed during latency 0 and I, presumably raising the bar for lytic reactivation to maintain persistence in long-lived memory B cells.

## Regulation of Host Transcripts by EBV ncRNAs

EBV miRNAs do not only compromise lytic EBV replication by targeting the viral DNA polymerase and immediate early transactivators of lytic EBV infection, but also by down-regulating cellular machinery that induces lytic EBV reactivation. BHRF1 miRNA 2-5p and BART miRNA 2-5p have been recently described to target components of B cell receptor signaling (Chen et al., 2019) which is involved in reactivating lytic EBV infection from latency I or 0 (Binne et al., 2002). The targeted transcripts encode GRB2, SOS1, MALT1, RAC1, and INPP5D, and the respective viral miRNAs attenuate lytic EBV reactivation upon BCR cross-linking. Furthermore, caspase 3 dependent apoptosis induction seems also to be required for lytic EBV replication and BART miRNA 20-5p was shown to down-regulate BAD, thereby compromising lytic replication (Lin et al., 2015; Kim et al., 2016). Furthermore, BART miRNAs 1-3p and 16 were reported to target caspase 3 directly (Vereide et al., 2014). Finally, the BART miRNA 18-5p down-regulates MAP kinase kinase kinase 2 (MAP3K2) and thereby also blocks lytic EBV infection (Qiu and Thorley-Lawson, 2014). In contrast, late lytic EBV infection is supported by the BHRF1 miRNA 1 targeting the ubiquitin ligase RNF4 that modifies SUMO-conjugated targets (Li et al., 2017). Expression of miRNA regulation resistant RNF4 attenuates infectious viral particle release. Therefore, multiple miRNAs block induction of lytic EBV infection by downregulating both viral and host factors involved in it, but then might support infectious viral particle production.

In addition to blocking lytic replication BART miRNAs have been also reported to sustain proliferation and block apoptosis. They do so in part by regulating the Wnt/β-catenin pathway. BART miRNA 10-3p has been shown to down-regulate the Dickkopf WNT Signaling Pathway Inhibitor 1 (DKK1) in EBV positive gastric carcinoma cell lines (Min and Lee, 2019). This enhances proliferation and migration of the respective tumor cells. Moreover, BART miRNA 1-3p was reported to down-modulate Disabled Homolog 2 (DAB2) that attenuates the Wnt/β-catenin pathway (Min et al., 2020). The resulting DAB2 down-regulation blocked apoptosis. Furthermore, also the Wnt/β-catenin inhibitor Adenomatous Polyposis Coli (APC) is down-regulated by BART miRNA 19-3p (Zhang et al., 2020). Finally, Mitogen-Activated Protein Kinase 4 (MAP2K4) is down-regulated by BART miRNA 22, stimulating β-catenin dependent transcription (Liu et al., 2019). Thus, BART miRNAs down-regulate inhibitors of the Wnt/β-catenin pathway to enhance proliferation, apoptosis resistance and migration of EBV associated carcinomas.

Additional pathways are targeted by mostly BART miRNAs to increase apoptosis resistance and migration of EBV infected cells. These include p53 mRNA down-regulation by BART miRNA 5-3p (Zheng et al., 2018). This regulation enhanced proliferation of gastric carcinoma cells *in vitro* and in a xenograft model in mice. In addition, ATM/ATR dependent DNA repair is augmented by BART miRNAs (Zhou et al., 2019). This increases radioresistance of nasopharyngeal carcinoma cells in a xenograft model. Finally, BART miRNA 2-5p targets RND3, a negative regulator of Rho signaling (Jiang et al., 2020). BART miRNA 2-5p expression in EBV negative nasopharyngeal carcinoma cells promoted their migration and metastasis formation in a xenograft model. Many of the studies that argue for a pro-proliferative, anti-apoptotic and migration promoting function of BART miRNAs have been performed with nasopharyngeal or gastric carcinoma cell lines and carcinogenesis *in vivo* was reported to be increased by BART miRNAs (Qiu et al., 2015), while EBV infection of B cells in mice with reconstituted human immune system components was not significantly altered by BART miRNA deficiency (Murer et al., 2019). However, increased lytic EBV replication in the absence of BART miRNAs might have been missed due to the B95-8 EBV strain that was used and the small as well as transient contribution of lytic EBV replication to viral loads in this model (Antsiferova et al., 2014). Nevertheless, an EBV strain lacking all viral miRNAs replicated to similar viral loads and similar lymphomagenesis in mice with reconstituted immune system components after antibody depletion of T cells (Murer et al., 2019). This suggests that immune escape might be the dominant function of EBV miRNAs, as discussed below.

Similar to their effects on viral gene products, EBER1 and 2 regulation of host gene products remains unclear. While these EBV ncRNAs trap RNA binding proteins like La (SS-B) and rpL22 in the nucleus (Fok et al., 2006; Gregorovic et al., 2011) the role of this relocalization has not been clarified. However, a recent study suggested that EBER1 can substitute for the ncRNA TMER4 in a mouse γ-herpesvirus to allow infected B cells to migrate from secondary lymphoid tissues into the circulation (Hoffman et al., 2019). Such changes in migratory behavior might not have been sufficiently analyzed during EBER deficient EBV infection of mice with reconstituted human immune system components, even so unaltered viral loads were observed in blood (Gregorovic et al., 2015).

## Immune Control of EBV

Although most of these pro-tumorigenic functions have been assigned to BART miRNAs of EBV, recombinant EBV that is deficient in BHRF1 miRNAs was found to infect mice with reconstituted human immune system components with a slower kinetic and attenuated systemic dissemination compared to wild-type virus (Wahl et al., 2013; Murer et al., 2019). This seems to be at least in part due to improved cell-mediated immune control (Murer et al., 2019).

Indeed, most adults carry EBV as an asymptomatic persistent infection (Münz, 2019). However, primary or acquired immunodeficiencies predispose for EBV associated malignancies (Damania and Münz, 2019; Latour and Fischer, 2019; Tangye and Latour, 2020). These point toward cytotoxic lymphocytes as the cornerstone of EBV specific immune control (Long et al., 2019). Immune suppression due to human immunodeficiency virus (HIV) co-infection or immune suppressive treatment after transplantation leads to increased EBV associated lymphoma formation (Gottschalk et al., 2005; Totonchy and Cesarman, 2016). This can be modeled by immunosuppressive FK506 treatment and HIV co-infection of EBV infected mice with reconstituted human immune system components (Caduff et al., 2020; McHugh et al., 2020). Both treatments compromise CD4^+^ T cell help leading to a less functional CD8^+^ T cell phenotype (Caduff et al., 2020; McHugh et al., 2020) and compromising CD8^+^ T cell mediated immune control of EBV infection with no further increase of viral loads upon antibody mediated depletion of this cytotoxic T cell subset (McHugh et al., 2020). Moreover, primary immunodeficiencies that predispose for EBV associated pathologies identify molecular requirements for EBV specific immune control. Mutations in perforin and gene products that mediate cytotoxic degranulation (Munc13-4 and 18-2) predispose for uncontrolled EBV infection and identify cytotoxicity as the main effector function during immune control of EBV (Katano et al., 2004; Rohr et al., 2010; Cohen et al., 2015). Furthermore, compromised T cell receptor signaling, predisposing for EBV associated pathologies, identifies these adaptive lymphocytes as main contributors to cell mediated immune control by cytotoxic lymphocytes (Huck et al., 2009; Linka et al., 2012; Angulo et al., 2013; Kuehn et al., 2013; Moshous et al., 2013; Lucas et al., 2014; Salzer et al., 2016; Hoshino et al., 2018; Winter et al., 2018). Accordingly, antibody mediated T cell depletion, and especially cytotoxic CD8^+^ T cell depletion, compromises immune control of EBV infection in mice with reconstituted human immune system components (Strowig et al., 2009; Yajima et al., 2009; Chijioke et al., 2015; Murer et al., 2019; McHugh et al., 2020). Furthermore, mutations in certain co-receptors on cytotoxic natural killer and T cells are also associated with EBV positive malignancies. This is particularly pronounced for deficiencies in CD27 and its ligand CD70 (Salzer et al., 2012; van Montfrans et al., 2012; Alkhairy et al., 2015; Abolhassani et al., 2017; Izawa et al., 2017; Ghosh et al., 2020). But also, mutations in the SLAM-associated protein (SAP), affecting co-stimulation of receptors like 2B4, predispose for EBV associated pathologies (Coffey et al., 1998; Nichols et al., 1998; Sayos et al., 1998; Sumegi et al., 2000; Pachlopnik Schmid et al., 2011). Accordingly, antibody blocking of 2B4 compromises EBV specific immune control in mice with reconstituted human immune system components but does not do so after CD8^+^ T cell depletion (Chijioke et al., 2015). In addition to cytotoxic CD8^+^ T cell mediated immune control also cytotoxic innate lymphocytes seem to contribute to EBV specific immune control. These include natural killer (NK) cells, NKT cells, and γδ T cells, controlling lytic and various stages of latent EBV infection, respectively (Pappworth et al., 2007; Yuling et al., 2009; Chijioke et al., 2013; Chung et al., 2013; Azzi et al., 2014; Xiang et al., 2014; Djaoud et al., 2017; Zumwalde et al., 2017). Therefore, cytotoxic 2B4^+^CD27^+^CD8^+^ T cells and innate lymphocytes seem to be crucial for EBV specific immune control, while type I and type II interferons as well as antibody responses appear to be dispensable (Latour and Fischer, 2019). Maybe not surprisingly EBV ncRNAs have therefore also a crucial function in modulating this immune control, as will be discussed next.

## Immune Modulation by EBV ncRNAs

Immune modulatory functions have been described for both EBERs and miRNAs. Indeed, when complete miRNA and BART miRNA knock-out viruses of the B95-8 EBV strain were compared for infection in mice with reconstituted human immune system components, no significant differences between wt and BART miRNA deficient EBV were detected, while complete miRNA deficient virus infection was significantly attenuated (Murer et al., 2019). Both viral loads and lymphomagenesis were lower with the miRNA deficient virus with no tumors detected in the absence of miRNAs. However, upon depletion of CD8^+^ T cells both viral loads and tumor formation were increased. More than 200-fold increased viral loads for miRNA deficient and more than 40-fold for wt EBV infection were detected after antibody mediated CD8^+^ T cell depletion, reaching similar levels for both infections. Lymphoma formation was also increased in frequency upon CD8^+^ T cell depletion with more than 50% of animals developing tumors, up from no tumors in miRNA deficient and 20–30% in wt EBV infected mice. These findings suggest that immune evasion from CD8^+^ T cell mediated immune control is one of the main functions of EBV miRNAs, especially the BHRF1 cluster.

Indeed, CD8^+^ T cell recognition of LCLs carrying miRNA deficient EBV is significantly increased (Albanese et al., 2016; Murer et al., 2019). This is associated with down-regulation of the transcripts for TAP2 (Figure 1), one of the two chains of the transporter associated with antigen processing that imports peptides into the endoplasmic reticulum (ER) for MHC class I loading and presentation to CD8^+^ T cells (Albanese et al., 2016). This diminished peptide import reduces particularly the surface expression of HLA-B molecules by which many immunodominant EBV derived epitopes are restricted (Taylor et al., 2015). TAP2 is targeted by the BHRF1 miRNA 3 and the BART miRNA 17 (Albanese et al., 2016). In addition, CD4^+^ T cell mediated immune control of LCLs is also compromised by viral miRNAs (Tagawa et al., 2016). CD4^+^ T cells recognize LCLs with deficiency in miRNA much better than wt EBV transformed B cells. MHC class II ligands for CD4^+^ T cell stimulation are primarily generated by lysosomal proteolysis (Trombetta and Mellman, 2005). EBV miRNAs downregulate some components of this degradation machinery, namely IFN-γ–inducible lysosomal thiol reductase (GILT) that breaks disulfide bonds prior to protein degradation, and the proteases cathepsin B and asparaginyl endopeptidase (AEP) (Figure 1; Tagawa et al., 2016). GILT is down-regulated by BART miRNAs 1–5p and 1–3p, cathepsin B by BART miRNA 2–5p, and AEP by BHRF1 miRNA 2 and BART miRNA 2–5p (Albanese et al., 2016; Tagawa et al., 2016). Moreover, also the cytokine IL-12 that is instrumental for T cell priming and activation of NK cells is targeted by viral miRNAs (Albanese et al., 2016; Tagawa et al., 2016). BHRF1 miRNA 2, BART miRNA 1–3p, BART miRNA 2–5p, BART miRNA 10-3p and BART miRNA 22 down-regulate IL-12p40 which is part of both IL-12 and IL-23 cytokines (Figure 1). NK cell recognition is further compromised by BART miRNA 2-5p and 7 down-regulation of MICB and MICA, respectively (Figure 1; Nachmani et al., 2009; Wong et al., 2018), two ligands of the activating NK cell receptor NKG2D which has been implicated in the recognition of lytically EBV replicating B cells (Pappworth et al., 2007). As additional immune escape mechanisms EBV miRNAs lead to the up-regulation of PD-L1 and PD-L2, ligands for the inhibitory receptor PD1 on cytotoxic lymphocytes (Cristino et al., 2019; Yoon et al., 2020). Thus, EBV miRNAs compromise immune control by cytotoxic lymphocytes on multiple levels, by targeting antigen presentation, immune stimulation and inhibitory receptor engagement, most likely for efficient virus persistence and transmission. Since, however, CD8^+^ T cell depletion restores viral load and lymphomagenesis of miRNA deficient EBV infection in mice with reconstituted human immune system components (Murer et al., 2019), regulation of MHC class I presentation might be one of the dominant functions of EBV encoded miRNAs.

EBERs have been suggested to also influence immune responses by being sensed via pathogen associated molecular pattern (PAMP) recognition receptors (PRRs) that detect viral RNAs, including toll-like receptor 3 (TLR3) and 8 (TLR8), RIG-I (Figure 1) and PKR (Vuyisich et al., 2002; McKenna et al., 2006; Samanta et al., 2008; Iwakiri et al., 2009; Li et al., 2019). Such immune stimulation by EBERs might also be transferred via EBER containing exosomes to dendritic cells (Ahmed et al., 2014, 2018; Baglio et al., 2016). BART miRNA 6-3p down-regulates this recognition by attenuating the RIG-I pathway (Figure 1; Lu et al., 2017). Despite this suggested role for innate immune recognition of EBERs, EBER deficient EBV infection of mice with reconstituted human immune system components did not result in a significantly altered immune activation (Gregorovic et al., 2015). The viral DNA is via TLR9 mediated detection at least an equally important immune stimulus and readily sensed by plasmacytoid dendritic cells (Fiola et al., 2010; Severa et al., 2013; Gujer et al., 2019). Thus, the human immune system does not seem to require EBERs for detection of EBV infection.

## Conclusion and Outlook

Non-coding RNAs are the ideal tools for viruses to modulate their life cycle, host cell behavior and immune responses, because they do not need the expression of proteins that could serve as antigens for the immune system to detect infection. Along these lines EBV extensively modulates its life cycle, promoting latency and suppressing lytic replication with BART miRNAs. Furthermore, its BHRF1 miRNAs seem to serve a crucial role in immune escape from cytotoxic lymphocytes, compromising anti-viral NK, CD4^+^ T, and CD8^+^ T cell responses. This realization offers the opportunity to explore miRNA deficient viruses, probably with additional safety mutations as vaccine candidates. Their superior ability to be recognized by both CD4^+^ and CD8^+^ T cell responses should be harnessed to induce both systemic and tissue resident T cell memory to protect from symptomatic acute EBV infection, namely infectious mononucleosis, and the associated risk for EBV positive malignancies and the autoimmune disease multiple sclerosis (MS) (Ruhl et al., 2020).

In contrast, EBERs remain an enigma of EBV biology. Their high expression in all EBV infection programs mocks us, having not identified a non-redundant function for these non-translated RNAs. It might require more sophisticated *in vitro* and *in vivo* systems of EBV infection and immune control to reveal the function of EBER1 and 2, possibly with more physiological migration behaviors of EBV infected cells from mucosal sites to secondary lymphoid organs (Hoffman et al., 2019). EBERs are joined by other non-translated RNA species, including small nucleolar RNAs (snoRNAs), long non-coding RNAs (lncRNAs), stable intronic sequence RNAs (sisRNAs) and circular RNAs (circRNAs), for which also functions need to be explored in more detail in the future. Nevertheless, it is already clear that EBV masterfully manipulates the cellular RNA network in addition to its fine-tuned proteins exploiting human B cell differentiation.

## Author Contributions

CM wrote the manuscript.

## Conflict of Interest

The author declares that the research was conducted in the absence of any commercial or financial relationships that could be construed as a potential conflict of interest.
